# Analysis of mitochondrial transcription factor A SNPs in alcoholic cirrhosis

**DOI:** 10.3892/etm.2013.1353

**Published:** 2013-10-21

**Authors:** CHUN TANG, HONGMING LIU, YONGLIANG TANG, YONG GUO, XIANCHUN LIANG, LIPING GUO, RUXIAN PI, JUNTAO YANG

**Affiliations:** Department of Hepatobiliary Surgery, Daping Hospital and Research Institute of Surgery, Third Military Medical University, Chongqing 400042, P.R. China

**Keywords:** alcoholic cirrhosis, mitochondrial transcription factor A, single nucleotide polymorphisms, mitochondrial DNA, susceptibility

## Abstract

Genetic susceptibility to alcoholic cirrhosis (AC) exists. We previously demonstrated hepatic mitochondrial DNA (mtDNA) damage in patients with AC compared with chronic alcoholics without cirrhosis. Mitochondrial transcription factor A (mtTFA) is central to mtDNA expression regulation and repair; however, it is unclear whether there are specific mtTFA single nucleotide polymorphisms (SNPs) in patients with AC and whether they affect mtDNA repair. In the present study, we screened mtTFA SNPs in patients with AC and analyzed their impact on the copy number of mtDNA in AC. A total of 50 patients with AC, 50 alcoholics without AC and 50 normal subjects were enrolled in the study. SNPs of full-length mtTFA were analyzed using the polymerase chain reaction (PCR) combined with gene sequencing. The hepatic mtTFA mRNA and mtDNA copy numbers were measured using quantitative PCR (qPCR), and mtTFA protein was measured using western blot analysis. A total of 18 mtTFA SNPs specific to patients with AC with frequencies >10% were identified. Two were located in the coding region and 16 were identified in non-coding regions. Conversely, there were five SNPs that were only present in patients with AC and normal subjects and had a frequency >10%. In the AC group, the hepatic mtTFA mRNA and protein levels were significantly lower than those in the other two groups. Moreover, the hepatic mtDNA copy number was significantly lower in the AC group than in the controls and alcoholics without AC. Based on these data, we conclude that AC-specific mtTFA SNPs may be responsible for the observed reductions in mtTFA mRNA, protein levels and mtDNA copy number and they may also increase the susceptibility to AC.

## Introduction

Alcoholic cirrhosis (AC) is an end-stage alcoholic liver disease (ALD) caused by long-term excessive drinking. It manifests as chronic inflammation and progressive fibrosis of the liver tissue and is the leading cause of mortality in chronic alcoholics. A 48-month prospective study of 280 patients with severe ALD in the United States revealed that 30% of the patients had alcoholic fatty liver disease, that more than half of those individuals developed cirrhosis and that two-thirds of the patients with cirrhosis developed alcoholic hepatitis and ultimately succumbed (1).

AC has a complex pathogenesis and has been indicated to be the result of the external environment (alcoholism) and genetic factors (2). Therefore, it is important to study the genetic components of AC to identify susceptible populations according to genotypes; such findings may be significant for the prevention and treatment of AC. At present, a number of genetic factors, including alcohol dehydrogenase, aldehyde dehydrogenase (3), apolipoprotein E (4) and cytochrome 450-2E1 (CYP2E1) gene polymorphisms (5,6), as well as superoxide dismutase gene dimorphisms (7), have been demonstrated to be associated with AC susceptibility. These genetic studies have focused on polymorphisms in enzymes involved in alcohol metabolism; there have been few studies on genetic factors affecting hepatocyte susceptibility to alcohol damage.

We previously demonstrated that hepatic mitochondrial DNA (mtDNA) in patients with AC exhibited reduced copy numbers, large deletions from bases 749 to 15,486 and a downregulation of one of its encoding products, cytochrome *c* oxidase 2. By contrast, no significant mtDNA damage was observed in chronic alcoholics without AC. The results suggested that specific mtDNA damage may be an important pathogenic factor in AC (8). mtDNA replication, transcription and repair are regulated by nuclear genes (9), primarily mitochondrial transcription factor A (mtTFA) (10) and nuclear respiratory factor 1 (NRF-1) (11). mtTFA is a nuclear-encoded factor that is central to mtDNA expression, regulation and repair (10). Therefore, we hypothesized that there may be mtTFA single nucleotide polymorphisms (SNPs) unique to patients with AC that affect the expression of mtTFA and its ability to repair damaged mtDNA in liver cells, thereby affecting normal mitochondrial functions and contributing to the pathogenesis of AC.

In this study, we aimed to screen SNPs in mtTFA in patients with AC and analyze their impact on hepatic mtDNA copy number and genetic susceptibility to AC. Our results are likely to enhance the understanding of AC pathogenesis and provide a new approach to the clinical prevention of the disease.

## Materials and methods

### Patients and clinical specimen collection

Study subjects were enrolled from Daping Hospital, Third Military Medical University (Chongqing, China), between June 2007 and June 2011. All experimental procedures were approved by the Medical Ethics Committee of the Third Affiliated Hospital of the Third Military Medical University. Written informed consent was obtained from each subject prior to specimen collection. Due to the fact that alcoholism is much more common in males than females in this region, all specimens were collected from male subjects. Blood (5 ml) was collected by venipuncture for liver function tests and mtTFA SNP analysis. Liver biopsies were collected for pathological examination and assessment of mtTFA expression and mtDNA copy number. AC was diagnosed according to the 2006 ALD diagnostic criteria established by the Chinese Medical Association (12), as follows: i) a long-term history of heavy drinking and alcohol intake ≥40 g/day for more than five consecutive years; ii) hypohepatia and portal hypertension, cirrhosis confirmed by imaging and alcoholic liver injury confirmed by a serum enzyme test; and iii) negative for hepatitis B and C antigens and antibodies and DNA tests (to exclude patients with cirrhosis due to other causes). Alcohol intake was calculated as follows: Alcohol intake = alcoholic drink intake (ml) × alcohol percentage × 0.8.

Three groups were assessed in this study. Group A (normal subjects): No long-term alcohol consumption, no liver diseases and no significant liver changes observed during upper abdominal surgery for other diseases (e.g. gallbladder stones). A total of 50 peripheral blood and 25 liver tissue specimens were collected from this group. Group B (chronic alcoholics without AC): Chronic alcoholism (confirmed according to the diagnostic criteria listed previously), normal liver function test results and no significant liver changes observed using imaging or during upper abdominal surgery for other diseases. A total of 50 peripheral blood and 26 liver tissue specimens were collected from this group. Group C (patients with AC): The three inclusion criteria described previously were met and liver scarring was confirmed during upper abdominal surgery for AC or other diseases. A total of 50 peripheral blood and 22 liver tissue specimens were collected from this group. For pathological examination, the liver specimens fixed in 10% formalin were embedded in paraffin. Sections of 4 μm were cut and stained with hematoxylin and eosin. Light microscopic evaluation was performed on the sections in a blinded fashion to assess liver parenchymal changes.

### Analysis of mtTFA SNPs

#### Genomic DNA extraction

A Universal Genomic DNA Extraction kit (Takara Biotechnology Ltd., Dalian, China) was used to extract total DNA from peripheral venous blood, in accordance with the kit’s instructions, and the extracted DNA was stored at −20°C.

#### Polymerase chain reaction (PCR) primers

Fifteen primers (Table I) were designed according to the full-length sequences of mtTFA (gi: 224589801) to perform segmented full-length PCR amplification of mtTFA.

#### PCR system and conditions

The Tiangen 2X Taq PCR MasterMix [Tiangen Biotech (Beijing) Co., Ltd., Beijing, China] was used for PCR amplification. The loading system included 1.5 μl template DNA, 1 μl each of forward and reverse primers (10 μM), 12.5 μl 2X Master Mix and 9 μl H_2_O. The total reaction volume was 25 μl. The amplification consisted of initial denaturation for 3 min at 94°C, 30 cycles of denaturation for 30 sec at 94°C, primer annealing for 30 sec at 58°C and primer extension for 1 min at 72°C, prior to primer extension for 5 min at 72°C after 30 cycles. A total of 5 μl PCR products were then mixed with 2.5 μl 6X loading buffer and the mixture was subsequently analyzed by 1.2% agarose gel electrophoresis (6 V/cm, 20 min).

All PCR products were purified prior to being submitted to Sangon Biotechnology Co., Ltd. (Shanghai, China) for sequencing. The resulting sequences were compared with genomic sequences of normal subjects in GenBank (http://www.ncbi.nlm.nih.gov/gene/7019) to identify SNPs in each group. Samples from the three groups were compared to screen for mtTFA SNPs unique to patients with AC. Gene analysis was performed using Cluxtal X (SNPStats, web tool for SNP analysis. http://bioinfo.iconcologia.net/snpstats/start.htm) and mtTFA SNP frequency was analyzed in patients with AC.

#### Detection of mtTFA expression in hepatocytes by quantitative PCR (qPCR)

Due to the fact that liver tissue was only collected from 22 patients with AC, we randomly selected 22 cases from the normal controls and chronic alcoholics without AC.

Total RNA was isolated from liver cells using guanidine isothiocyanate extraction and randomly selected samples were evaluated using electrophoresis. Portions of the samples were reverse transcribed into cDNA using PrimeScript Reverse Transcriptase (Takara Biotechnology Ltd.), in accordance with the manufacturer’s instructions. The PCR primers used were as follows: mtTFA forward, 5′-AGATTCCAAGAAGCTAAG GGTGATT-3′ and reverse, 5′-TTTCAGAGTCAGACAGAT TTTTCCA-3′; β-actin forward, 5′-GATGACCCAGATCAT GTTTGAG-3′ and reverse, 5′-AGGGCATACCCCTCGTA GAT-3′. SYBR Green qPCR Master Mix (2X) from Ruian Biotechnologies Co., Ltd. (Shanghai, China) and the ABI 7500 Fast Real-Time PCR system (Applied Biosystems, Life Technologies, Carlsbad, CA, USA) were used for qPCR. Amplification was performed in 25-μl reaction mixtures containing 12.5 μl SYBR Green qPCR Master Mix, 0.5 μl each of forward and reverse primers (10 μM), 9.5 μl ddH_2_O and 2 μl template (cDNA). The following cycling protocol was used: 2 min at 95°C, followed by 40 cycles consisting of 10 sec at 95°C and 40 sec at 60°C. The relative quantitative mtTFA value was represented by the ratio of mtTFA to β-actin. Three parallel experiments were conducted for each sample.

#### Western blot analysis of mtTFA in liver tissue

Denatured protein samples (50 mg) were subjected to sodium dodecyl sulfate-polyacrylamide gel electrophoresis (SDS-PAGE), transferred under semi-dry conditions onto nitrocellulose (NC) membranes and visualized using Ponceau-S staining. Following this, the NC membranes were blocked with Tris-buffered saline solution (TBS) containing 5% non-fat dried milk overnight, prior to being incubated with mtTFA antibody (1:400 dilution) at 4°C overnight and washed in TBS with Tween (TBST) three times (10 min each). The membranes were subsequently incubated with goat anti-rabbit immunoglobulin (Ig) G (PICPI31462 - Pierce Peroxidase Affinity-Purified Polyclonal Antibodies - HRP, Thermo Fisher Scientific Inc., Waltham, MA, USA; 1:5,000 dilution) at room temperature for 1 h and washed a further three times in TBST (10 min each). The blots were visualized using enhanced chemiluminescence substrate (PICPI32209 - Pierce ECL Western Blotting Substrate, Thermo Fisher Scientific Inc.) and X-ray films. Bands were analyzed according to their optical density and the values were normalized to β-actin bands.

#### Detection of hepatocyte mtDNA copy number by qPCR

A Universal Genomic DNA Extraction kit (Takara Biotechnology Ltd.) was used to extract total DNA from the liver tissues, in accordance with the kit’s instructions. The extracted DNA was stored at −20°C.

Due to the fact that the mtDNA displacement (D)-loop region is highly conserved, it was used as a surrogate for the mtDNA copy number. The nuclear β-globin served as an internal control. The PCR primers used were as follows: mtDNA forward, 5′-TTGCACGGTACCATAAATACTTGAC-3′ and reverse, 5′-GAGTTGCAGTTGATGTGTGATAGTTG-3′; β-globin forward, 5′-CAACTTCATCCACGTTCACC-3′ and reverse, 5′-CAACTTCATCCACGTTCACC-3′. The hepatic mtDNA copy number was determined using qPCR with a SYBR^®^ Premix Ex Taq™ II (Perfect Real-Time) kit from Takara Biotechnology Ltd. The PCR system included 12.5 μl SYBR^®^ Premix Ex Taq™, 2 μl DNA template (~100 ng), l μl each of forward and reverse primers (final concentration, 0.4 μmol/l) and 8.5 μl H_2_O. The total reaction volume was 25 μl. qPCR was performed using a Bio-Rad CFX96 real-time PCR system (Bio-Rad, Hercules, CA, USA) at conditions of 95°C for 30 sec, followed by 40 cycles of 95°C for 5 sec, 55°C for 30 sec and 72°C for 30 sec. The relative quantitative mtTFA value was represented by the ratio of mtTFA to β-globin. Three parallel experiments were conducted for each sample.

#### Statistical analysis

All data are presented as the mean ± standard deviation (SD). Groups were compared using one-way analysis of variance and Student-Newman-Keuls tests, with P<0.05 considered to indicate a statistically significant difference.

## Results

### Study subject clinical characteristics

Table II summarizes the clinical and biochemical characteristics of the three groups. There were no significant differences in age among the three groups or in the duration of alcoholism (years) or daily intake of alcohol (g) between groups B and C. The patients in group C had significantly impaired liver function, as evidenced by increased levels of total bilirubin and aspartate aminotransferase (AST) and the decreased albumin level (Table II). Among the patients with AC, there were 36 (72%) with a history of upper gastrointestinal bleeding, 23 (46%) with ascites and 26 (52%) with hypersplenism.

Eighteen patients underwent splenectomy and pericardial devascularization and two patients in group C underwent laparoscopic cholecystectomy during the liver function compensation stage. A further two patients in group C received emergency splenectomy and pericardial devascularization due to upper gastrointestinal bleeding that was not controlled by conservative non-surgical treatment. In groups A and B, 25 and 26 patients, respectively, underwent laparoscopic cholecystectomy. Liver tissue specimens were collected during surgery.

### Pathological examination

**Figure 1:**
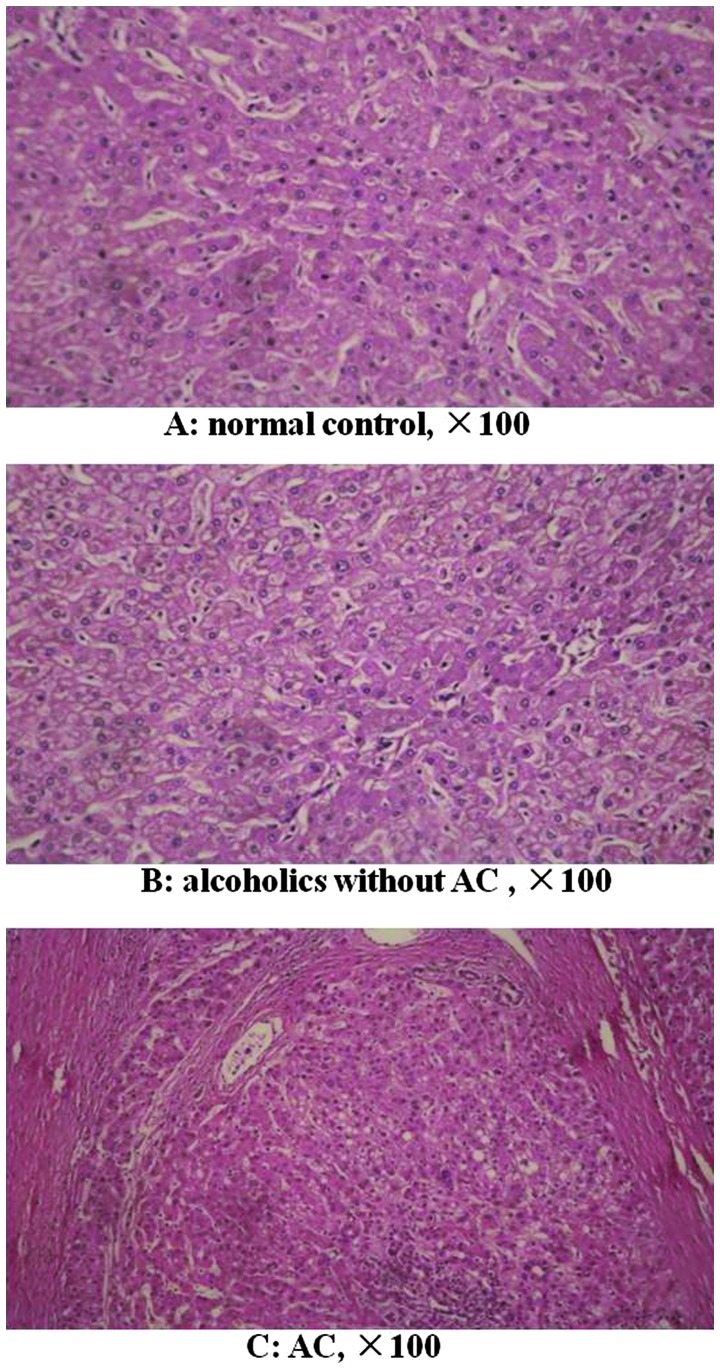
Histopathological liver findings by hematoxylin and eosin staining. (A) Normal control group. (B) Alcoholics without alcoholic cirrhosis (AC) group (magnification, ×100): None of the patients showed any morphological cirrhotic changes. (C) AC group (magnification, ×100): all patients showed morphological changes and typical pseudolobule formation.

All liver tissue specimens were pathologically examined. There were no morphological cirrhotic changes in the specimens from the normal control and alcoholics without AC groups (Fig. 1A and B). In the normal control group, certain patients showed a normal morphological liver tissue structure, while a number had mild steatosis. In the alcoholics without AC group, certain patients exhibited a normal morphological liver tissue structure, while steatosis was observed in others. In the AC group, all patients showed morphological changes in the liver tissue, including typical pseudolobule formation (Fig. 1C).

### SNPs unique to patients with AC

The three groups were compared to screen 72 base mutations unique to group C that were not present in groups A and B. Among them, 18 SNPs occurred in patients with AC at frequencies >10% (Table III).

Table III shows that mtTFA SNPs were present from bases 664 to 678, 2,542 to 3,361, 4,985 to 5,223 and at base 7,648 in patients with AC. Among the 18 SNPs present in the full-length 10,722-bp mtTFA, seven were known in the SNP Database (dbSNP) and 11 were newly discovered. According to the mtTFA gene information provided by GenBank, the SNPs at 3,278 and 3,361 were located in the same coding region, while the remaining SNPs were located in non-coding regions.

### mtTFA SNPs unique to patients with AC and normal subjects

We also screened 28 base mutations unique to groups A and C that were not present in group B. Five mutations occurred in group C at frequencies >10% (Table IV); one was in the dbSNP and four were novel. Due to the fact that group A did not have a long-term history of alcohol abuse, if the individuals with these five mutations in group A were to develop AC following alcohol abuse, these mtTFA SNPs may be considered to be a contributing factor to AC. If the individuals were not to develop AC, it may be that these SNPs have other genetic influences. While our results suggest that these five mtTFA SNPs may be specific to AC, further investigations are required.

### mtTFA SNP distribution in patients with AC

The number of unique mtTFA SNPs was calculated to be between zero and six in each patient with AC. It was determined that 70% had between two and four SNPs (Table V).

### Analysis of mtTFA mRNA expression using qPCR

**Figure 2:**
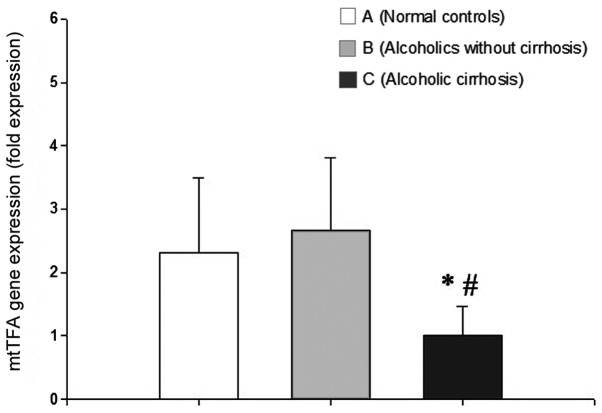
Alterations in mitochondrial transcription factor A (mtTFA) mRNA expression in liver tissue in: (A) normal controls, (B) alcoholics without alcoholic cirrhosis (AC) and (C) patients with AC. Data are presented as the mean ± standard deviation (n=22/group) and were compared with Student’s t-tests. ^*^P<0.05 vs. normal controls; ^#^P<0.05 vs. alcoholics without AC.

No significant difference was identified in the mtTFA mRNA level between the normal control and alcoholics without AC groups (P>0.05). By contrast, mtTFA mRNA expression was significantly lower in the AC group than in the normal control and the alcoholics without AC groups (P<0.05 for each; Fig. 2).

### Analysis of mtTFA protein expression using western blotting

**Figure 3:**
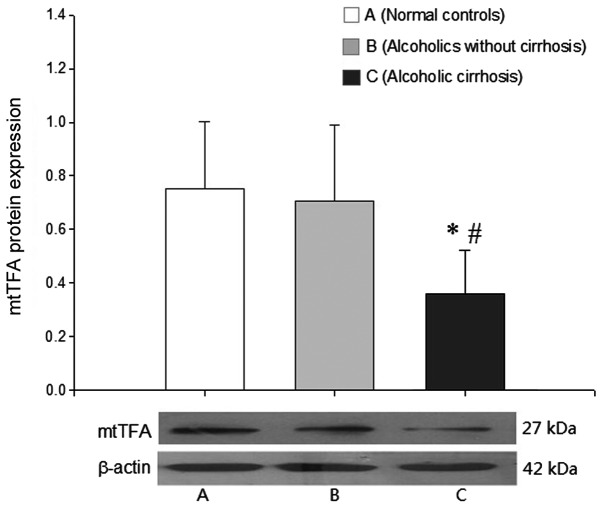
Alterations in mitochondrial transcription factor A (mtTFA) protein expression in liver tissue in: (A) normal controls, (B) alcoholics without alcoholic cirrhosis (AC) and (C) patients with AC. Data are presented as the mean ± standard deviation (n=22/group) and were compared with Student’s t-tests. ^*^P<0.05 vs. normal controls; ^#^P<0.05 vs. alcoholics without AC.

Western blot analysis did not demonstrate a significant difference in the level of mtTFA protein expression between the normal control and the alcoholics without AC groups. By contrast, the levels of mtTFA protein expression in the AC group were significantly decreased compared with those in the other two groups (P<0.05 for each; Fig. 3).

### Analysis of mtDNA copy number using qPCR

**Figure 4:**
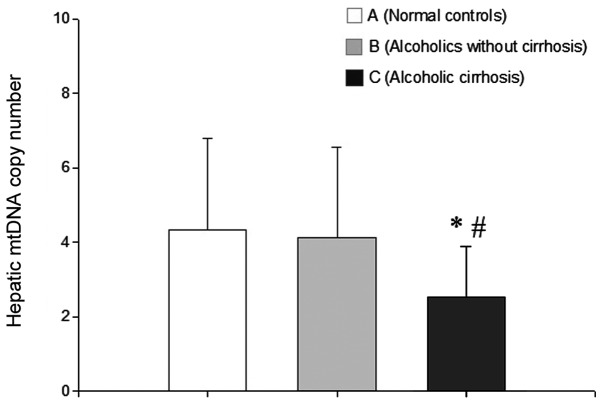
Alterations in hepatic mitochondrial DNA (mtDNA) copy number in: (A) normal controls, (B) alcoholics without alcoholic cirrhosis (AC) and (C) patients with AC. Data are presented as the mean ± standard deviation (n=22/group) and were compared with Student’s t-tests. ^*^P<0.05 vs. normal controls; ^#^P<0.05 vs. alcoholics without AC.

No significant difference in mtDNA copy number was identified between the normal control and alcoholics without AC groups. However, the mtDNA copy number was significantly lower in the AC group than in the normal control and the alcoholics without AC groups (P<0.05 for each; Fig. 4).

## Discussion

mtTFA is a nuclear-encoded factor that exerts powerful effects on mtDNA; it promotes mtDNA transcription and expression (13), increases mtDNA copy number (14), maintains integrity and stability (15), protects and repairs damaged mtDNA and restores mitochondrial function (16). Therefore, mtTFA genetic variation is likely to affect mtDNA number, structure and repair, thereby altering mitochondrial structure and function. As a result, this affects the extent of ethanol-induced liver cell damage.

SNPs are DNA sequence variations that occur when a single nucleotide differs among members of a biological species. Since it was first described in 1994 (17), SNP analysis has become a focus in fields that assess molecular markers. As a third generation genetic marker, SNPs are characterized by their high density and conservedness in the genome. Statistically, an SNP occurs every kilobase and it is estimated that there are more than three million SNPs in the entire three billion-base human genome. The majority of SNPs are located in non-coding regions; however certain SNPs are present in gene promoters and are thus able to increase or decrease gene transcription activity, thereby affecting protein expression and biological activity (18). A number of SNPs located in protein coding regions may change the amino acid sequence of key functional groups, which may subsequently affect protein function (19) and ultimately influence susceptibility to specific environments or pathogens.

The 10,722-bp human mtTFA gene is located on chromosome 10. Based on the estimated frequency of SNPs, it is predicted that there are ~10 SNPs in the mtTFA gene. If certain SNPs affect normal mtTFA expression, they may also affect mitochondrial function via their effects on mtDNA, thus leading to cell dysfunction and damage. A genetic analysis of cattle revealed that specific SNPs in the mtTFA gene promoter region caused a decline in mtDNA copy number (20), while certain SNPs in cattle mtTFA affected animal growth and development (21,22). Clinical studies have demonstrated that mtTFA SNPs may cause a shortage of intracellular mtDNA copies, which is a risk factor for Alzheimer’s disease (23–25). However, the existence of specific mtTFA SNPs in human patients with AC has not been described, to date.

In this study, 18 mtTFA SNPs were identified in 50 patients with AC, including five possible AC-specific SNPs. These SNPs occurred at a frequency of 0.168%, which is significantly higher than ~0.1% in the normal population. Two of the SNPs were located in the same coding region and were likely to affect normal mtTFA expression. Although the remaining SNPs were located in non-coding regions, they were present at particular base sequences. Two were located at bases 664 to 678 (total length of 14 bp), eight were located at bases 2,542 to 2,930 (total length of 389 bp) and five were at bases 4,985 to 5,223 (total length of 239 bp). SNPs occurring in such short base sequences at such a high frequency are also likely to affect normal mtTFA expression, despite the fact that they are in non-coding regions. In addition, the analysis of mtTFA SNP distribution in patients with AC demonstrated that these SNPs were not present in five patients (10%), suggesting that the underlying causes of AC are complex and multifactorial (3–7).

mtTFA mRNA and protein levels were measured in liver tissue using qPCR and western blotting, respectively, to investigate whether mtTFA SNPs in patients with AC affected mtTFA expression. The results showed that mRNA and protein levels were significantly lower in the AC group than in the normal control and alcoholics without AC groups (P<0.05 for each). Appropriate mtTFA expression is required for mtDNA stability and mtTFA protein level directly affects mtDNA copy number (26). Decreased expression of mtTFA reduces the ability to repair mtDNA damage and results in a consequent reduction in the mtDNA copy number. Notably, it was observed that the mtDNA copy number was significantly reduced in patients with AC.

There are a number of reasons why long-term alcohol abuse may result in mtDNA damage. Oxidative stress is believed to be important in AC pathogenesis and development, and mitochondria are particularly vulnerable to oxidative stress (27). Excessive oxidative stress injury may induce apoptosis, the main mechanism of progressive hepatic injury, ultimately leading to cirrhosis (28). Furthermore, the liver is the organ that is primarily responsible for metabolizing ethanol. Hepatocytes oxidize ethanol to acetaldehyde via alcohol dehydrogenase, subsequently producing acetic acid via acetaldehyde dehydrogenase and ultimately carbon dioxide and water (29). A high plasma ethanol concentration following heavy drinking may also activate the microsomal ethanol oxidizing system (MEOS), which catalyzes acetaldehyde production. However, ethanol-induced MEOS activity fails to oxidize ethanol to produce ATP and also increases oxygen and nicotinamide adenine dinucleotide phosphate (NADPH) consumption. This results in cell hypoxia and increased levels of oxygen free radicals (29), which are the most important factor causing mtDNA damage. In addition, acetaldehyde produced from ethanol metabolism may damage the antioxidant defense system and directly bind to DNA, thus inhibiting its repair (30).

The factors mentioned previously may all lead to hepatic mtDNA damage. Specific mtTFA SNPs may decrease mtTFA expression, resulting in a reduced ability to effectively repair mtDNA damaged by chronic alcoholism and a reduction in hepatic mtDNA copy number. Moreover, we previously demonstrated that hepatic mtDNA in patients with AC exhibited reductions in copy number and large deletions, in addition to a downregulation of one of its encoding products (8).

In conclusion, specific mtTFA SNPs may decrease mtTFA expression, resulting in an inability to effectively repair mtDNA damaged by chronic alcoholism. These changes may increase susceptibility to AC.

## Figures and Tables

**Table I:** Primer sequences for the segmented full-length polymerase chain reaction (PCR) amplification of mitochondrial transcription factor A (mtTFA).

No.	Forward	Reverse
1	TAC CTT CGA TTT TCT AAA	TCA ACA AAC ATC CTA CCT
2	GAT AAA TCC TTT CTT GTC T	AAT ACA TTC TCG ATC CAT
3	CGG TTA AGA TGA AGA AGG	CCA ACT AAT TTA AAC GTA AGT A
4	TAC TCC TTA GTT GTT AGA T	AGT AAG TCA ACA AAC CAT
5	AGA GCA CAT TTT CCA CCT	CCA TAT CAA ACT CAC CAT
6	TGA AAC AGT CAA ACC AGG AG	GGG AGA ATG AAA GAG GGA
7	GGA GCC AGA AAG ATA CTA	TTT AAA GCT CCA TAG TTG
8	AAT GCA ATA TCA CTC CCT	GAA TCA CCC TTA GCT TCT
9	CAG GAA AAG TCT AGA GTG	AAA TCA AGA AGC AAC AAT
10	TTC CAT ATC CCT AAA TAA C	CAA TAA ATC CCT ATA CCT T
11	GTA AAG GTG CAT GGG AGA	AAT TAG CCA GGC TTG GTG
12	CAA CCT CTT GAG TAG CCA	TGA AAA GCA GAT GCA TTA
13	ACA ATT AGC TTT CTT TTG TC	CTG TGC TTC AGT GTT TAG
14	ATA AAA CCT AAA GCT ACA	GAA TGA AAT TGT TAC AAG T
15	TTC TCC AGT CTG CCT TTA	CCT TTA TCT GGG TTT TCC

**Table II:** Clinical and biochemical characteristics of the study subjects.

Characteristic	Group A (Normal controls)	Group B (Alcoholics without AC)	Group C (AC)
Number of cases	50	50	50
Gender	Male	Male	Male
Age (years)	50.35±11.96 (34–81)	48.78±11.82 (30–73)	52.98±12.13 (33–83)
Average daily alcohol intake (g)	-	109.80±44.65	114.40±55.11
Years of alcoholism	-	24.90±10.08	25.80±12.26
Total bilirubin (μmol/l)	18.32±9.74	17.78±7.60	34.32±16.43^a^
ALT (U/L)	35.75±13.02	35.78±19.64	29.41±16.10
AST (U/L)	36.86±14.83	37.74±12.50	49.85±12.48^a^
Albumin (g/l)	38.16±5.32	37.45±5.88	30.75±6.06^a^

a P<0.05 versus normal controls or alcoholics without alcoholic cirrhosis (AC).

ALT, alanine aminotransferase; AST, aspartate aminotransferase.

**Table III:** SNPs of mtTFA unique to patients with AC (frequency >10%).

Locus	Mutation	Number	Frequency (%)	dbSNP
664	G-A	9	18	
678	G-A	7	14	
2542	A-G	5	10	
2557	C-T	9	18	
2582	G-A	16	32	
2596	A-T	5	10	rs189223626
2601	A-G	7	14	rs139675989
2641	G-A	5	10	
2665	C-G	5	10	rs193210579
2930	A-C	8	16	rs145188595
3278	G-A	11	22	rs139514719
3361	T-G	5	10	
4985	A-T	10	20	
5068	G-T	5	10	rs140714664
5106	A-G	5	10	
5217	C-T	7	14	rs19060687
5223	C-A	8	16	
7648	A-T	6	12	

SNP, single nucleotide polymorphism; mtTFA, mitochondrial transcription factor A; AC, alcoholic cirrhosis; dbSNP, SNP Database reference.

**Table IV:** mtTFA SNPs unique to patients with AC and normal subjects (frequency >10%).

		Number		Frequency (%)		
				
Locus	Mutation	Group A	Group C	Group A	Group C	dbSNP
680	G-A	2	6	4	12	
2344	C-T	7	9	14	18	rs184602713
2652	A-G	5	10	10	20	
3261	G-T	2	7	4	14	
6836	C-G	7	5	14	10	

SNP, single nucleotide polymorphism; mtTFA, mitochondrial transcription factor A; AC, alcoholic cirrhosis; dbSNP, SNP Database reference.

**Table V:** Specific mtTFA SNP distributions in patients with AC (n=50).

Number of SNPs	Number of cases (%)
0	5 (10)
1	6 (12)
2	13 (26)
3	11 (22)
4	11 (22)
5	2 (4)
6	2 (4)
Total	50 (100)

SNP, single nucleotide polymorphism; mtTFA, mitochondrial transcription factor A; AC, alcoholic cirrhosis.
